# Editorial: Food allergy: advances in basic and translational animal models

**DOI:** 10.3389/falgy.2023.1299113

**Published:** 2023-10-10

**Authors:** Joseph J. Dolence, James W. Krempski, Jessica J. O’Konek

**Affiliations:** ^1^ Department of Biology, University of Nebraska at Kearney, Kearney, NE, United States; ^2^ Department of Pathology, University of Michigan, Ann Arbor, MI, United States; ^3^ Mary H Weiser Food Allergy Center, University of Michigan, Ann Arbor, MI, United States

**Keywords:** food allergy, mouse models, tolerance, microbiome, trained immunity, neuroimmune axis

**Editorial on the Research Topic** Food allergy: advances in basic and translational animal models

## Introduction

Food allergy is an adverse reaction to food driven by the generation of food-specific IgE and type 2 T cell-mediated immune responses (1, 2). Increasing prevalence in children and adults have made food allergy a significant concern for public health. Despite this, therapeutic options for patients with food allergies remain limited. Therefore, research to (1) increase the knowledge of the mechanisms of disease and (2) develop treatments to suppress established disease are of critical importance. Mouse models have made significant contributions to our understanding of food allergy, including immunological mechanisms of sensitization and reactivity and the preclinical development of therapeutics. A major focus of food allergy research is on increasing the knowledge of current models and refining and developing new animal models to closely replicate human disease.

This Frontiers Research Topic represents a collection of articles from laboratories specializing in mouse models of food allergy and contains novel studies describing the use of mouse models of cow's milk, wheat, egg, and peanut allergy. These articles highlight important considerations for mouse modeling of food allergy. They demonstrate both the usefulness of animal modelling in understanding of mechanisms of food allergy and anaphylaxis, as well as utility of these models in the preclinical development of therapeutics for food allergies.

## Mouse models of food allergy

Multiple models of food allergy have been described in the literature. The most commonly used models involve oral or systemic sensitization with allergen and an adjuvant or epicutaneous sensitization of tape-stripped skin (3, 4). Inhalation-based models have also demonstrated an ability to drive food allergic immune responses (5). These models have provided valuable insights into the mechanisms of food allergy. However, significant research is ongoing to develop new models that more closely recapitulate human disease. Gao et al. describe a novel model of wheat allergy driven by sensitization through intact skin without the use of adjuvants. Nine weekly applications of a protein extract from wheat onto intact skin resulted in sensitization, as characterized by increases in both wheat-specific IgE and total IgE and induction of a Th2 immune response. This sensitization was sufficient to induce reactivity to oral wheat challenge, including clinical symptoms as well as mast cell degranulation. This model may provide insights into the development of other mouse models of food allergies that do not rely on adjuvants or tape-stripping of the skin to induce a significant response and may be more clinically relevant.

## Factors that drive heterogeneity of allergic reactions

While allergen-specific IgE is a key component to diagnosis of food allergies, it is not solely predictive of whether a patient will react to the food or the degree of severity their reactions will be. It is clear that other factors are involved in determining if a sensitized individual will experience mild reactions or severe anaphylaxis or will be tolerant to the food. Several articles in this Research Topic underscore the heterogeneity of reactions to food and demonstrate factors that influence reactivity in mouse models. Stark et al. identifies the role of microbiome in regulating the severity of reactions in models of egg allergy. They demonstrate how antibiotic-mediated dysbiosis, including increased colonization by *Candida albicans* and loss of *Lachnoclostridium* species, enhances manifestations of allergy reactivity. Levels of allergen-specific IgE in the sera were not correlative with disease severity. These data recapitulate the observation in humans that levels of food-specific IgE do not correlate with severity of reactions. However, the strength of Th2 immune responses, as well as accumulation of mucosal mast cells correlated with severity of reactions. Interestingly, they found that genetically identical mice treated under the same protocols but housed in different rooms in the vivarium developed different levels of Th2 responses to the allergen, and this correlated with disease severity. They identified differences in intestinal microbiomes of the mice housed in different rooms as a cause of the heterogeneity of the severity of allergic reactions. In addition to highlighting a role for the microbiome in driving the severity of allergic reactions, this report is very important for researchers using animal models of food allergy, as it highlights how variability in the microbiome of an animal driven by a change in environment can lead to different results between investigators. The reliability and reproducibility of these models is an important goal for researchers. Since allergic responses to food in humans are more heterogenous than those found in animal models, future studies aimed at elucidating such differences will be critical as we grow our understanding of the mechanisms driving food allergy.

Germundson et al. investigated a potential genetic mechanism to explain differential severity of allergic reactions in individuals sensitized to foods. They investigated the role of HLA-II variants as genetic determinants influencing variability in food allergy symptoms in a mouse model of cow's milk allergy. HLA-II molecules on antigen presenting cells are critical for presenting antigen and therefore may play a role in initiating sensitization as well as the allergic response. The HLA-II gene is highly polymorphic, and allelic variations associated with increased susceptibility to egg and cow's milk allergy have been identified in humans (6, 7). Transgenic mice expressing different HLA-II alleles demonstrated that mice with certain HLA-II variants were largely asymptomatic upon oral challenge, while mice expressing other HLA-II variants had moderate to severe anaphylaxis. Taken together, these reports highlight that genetic and microbial factors influence the severity of allergic reactions, further supporting the knowledge that allergic reactions to food are due to complex interactions between a multitude of factors.

## Behavioral and neurological responses to food allergens

While the majority of food allergy research thus far has focused on immunological assays and readouts of reactivity, interest in the interface between the immune and nervous systems has increased in recent years, as evidence of bidirectional interactions of these distinct systems have been uncovered (8). Two papers in this Research Topic document neurological and behavioral changes in sensitized mice following oral food challenge. Brishti et al. utilized a mouse model of cow's milk allergy with demonstrated sensitization that results in non-anaphylactic responses to oral challenge, allowing for repeated exposure to allergen. Mice in this model that were sensitized to cow's milk and then fed a whey protein diet displayed neurological and behavioral symptoms, including anxiety-like behavior and spatial memory decline. These changes were associated with increased expression of chemokines responsible for leukocyte recruitment in the brain which led to persistent neuroinflammation. Germundson et al. also demonstrated that allergic reactivity was also associated with similar behavioral changes. These reports highlight the importance of further studies investigating the influence of food allergy on the brain and the ways in which the nervous and immune systems interact to influence disease.

## Animal models of food allergy for preclinical drug development

Another advantage of animal models is the ability to use them to test novel therapeutics. Mouse models have provided key data that was used to support clinical trials for treatments for allergic disease, including epicutaneous immunotherapy and biologics (9, 10). Hughes et al. utilized a mouse model of peanut allergy for preclinical studies of a novel nanoparticle approach for allergen-specific immunotherapy. They demonstrated that encapsulation of peanut extract in poly(lactic-co-glycolic acid) (PLGA) nanoparticles allowed for safe administration, compared with administration of free peanut extract which induced severe anaphylaxis. Prophylactic administration of these peanut nanoparticles prevented sensitization, and therapeutic administration suppressed reactivity to oral peanut challenge. Suppression of reactivity to peanut challenge was associated with reduced Th2 cytokine production in these mice, providing proof-of-concept data for the ability of nanoparticles to induce tolerance. Similar peanut nanoparticles are currently being tested in a clinical trial (NCT05250856).

## Role of trained innate immunity in driving food allergy

The review by Arzola-Martínez et al. reviewed the literature to present current knowledge of microbial, epigenetic and metabolic changes in the prenatal and neonatal period that influence immune development by driving trained innate immunity. Multiple environmental factors have been demonstrated to influence trained immunity, including the microbiome and vaccination as well as parasitic, bacterial, viral, or fungal infections. These factors can influence immune responses later in life and may be one component of predisposing an individual to develop food allergies. Alternatively, trained innate immunity could be a target for novel therapeutics to promote tolerance to treat or prevent food allergies. This review discusses how data from animal models and human trials have been critical for our current understanding and highlights how mouse models will be useful in providing further insights into the role of environmental factors on driving allergic disease.

Taken together, this Research Topic on animal models of food allergy provides a valuable collection that gives insight into the many ways mouse models enhance our understanding of food allergy and preclinical development of therapeutics.

## References

[1] Ramsey N Berin MC. Pathogenesis of IgE-mediated food allergy and implications for future immunotherapeutics. Pediatr Allergy Immunol. (2021) 32:1416–25. 10.1111/pai.13501 33715245 PMC9096874

[2] Lee D Krishnaswamy JK Gowthaman U. Common and distinct roles for T(H)2 and T(FH) cells in shaping the spectrum of allergic diseases. J Allergy Clin Immunol. (2022) 150:1050–2. 10.1016/j.jaci.2022.09.017 36174849

[3] Benede S Berin MC. Applications of mouse models to the study of food allergy. Methods Mol Biol. (2021) 2223:1–17. 10.1007/978-1-0716-1001-5_1 33226583

[4] Smeekens JM Kulis MD. Mouse models of food allergy in the pursuit of novel treatment modalities. Front Allergy. (2021) 2:810067. 10.3389/falgy.2021.810067 35387036 PMC8974753

[5] Kulis MD Smeekens JM Immormino RM Moran TP. The airway as a route of sensitization to peanut: an update to the dual allergen exposure hypothesis. J Allergy Clin Immunol. (2021) 148:689–93. 10.1016/j.jaci.2021.05.035 34111450 PMC8429226

[6] Savilahti EM Ilonen J Kiviniemi M Saarinen KM Vaarala O Savilahti E. Human leukocyte antigen (DR1)-DQB1*0501 and (DR15)-DQB1*0602 haplotypes are associated with humoral responses to early food allergens in children. Int Arch Allergy Immunol. (2010) 152:169–77. 10.1159/000265538 20016199

[7] Dimitrov I Doytchinova I. Associations between milk and egg allergens and the HLA-DRB1/DQ polymorphism: a bioinformatics approach. Int Arch Allergy Immunol. (2016) 169:33–9. 10.1159/000444172 26953725

[8] Kim B Rothenberg ME Sun X Bachert C Artis D Zaheer R Neuroimmune interplay during type 2 inflammation: symptoms, mechanisms and therapeutic targets in atopic diseases. J Allergy Clin Immunol. (2023). 10.1016/j.jaci.2023.08.017 [Epub ahead of print]. PMC11215634 37634890

[9] Mondoulet L Dioszeghy V Vanoirbeek JA Nemery B Dupont C Benhamou PH. Epicutaneous immunotherapy using a new epicutaneous delivery system in mice sensitized to peanuts. Int Arch Allergy Immunol. (2011) 154:299–309. 10.1159/000321822 20962535

[10] Le Floc’h A Allinne J Nagashima K Scott G Birchard D Asrat S Dual blockade of IL-4 and IL-13 with dupilumab, an IL-4Ralpha antibody, is required to broadly inhibit type 2 inflammation. Allergy. (2020) 75:1188–204. 10.1111/all.14151 31838750 PMC7317958

